# Toward better understanding of postharvest deterioration: biochemical changes in stored cassava (Manihot esculenta Crantz) roots:

**DOI:** 10.1002/fsn3.303

**Published:** 2015-10-26

**Authors:** Virgílio Gavicho Uarrota, Eduardo da Costa Nunes, Luiz Augusto Martins Peruch, Enilto de Oliveira Neubert, Bianca Coelho, Rodolfo Moresco, Moralba Garcia Domínguez, Teresa Sánchez, Jorge Luis Luna Meléndez, Dominique Dufour, Hernan Ceballos, Luis Augusto Becerra Lopez‐Lavalle, Clair Hershey, Miguel Rocha, Marcelo Maraschin

**Affiliations:** ^1^ Plant Science CenterPlant Morphogenesis and Biochemistry LaboratoryPostgraduate Program in Plant Genetic ResourcesFederal University of Santa CatarinaRodovia Admar Gonzaga 1346CEP 88.034‐001FlorianópolisSCBrazil; ^2^ Santa Catarina State Agricultural Research and Rural Extension Agency (EPAGRI)Experimental Station of Urussanga (EEUR)Rd. SC 446Km 19 S/NUrussangaFlorianópolisSCCEP 88840‐000Brazil; ^3^ International Center for Tropical Agriculture (CIAT)Apartado Aéreo 6713CaliColombia; ^4^ Centre de Coopération Internationale en Recherche Agronomique pour le Développement (CIRAD)UMR Qualisud73 Rue Jean‐Francois BretonTAB‐95/1634398Montpellier Cedex 5France; ^5^ Centre of Biological EngineeringUniversity of MinhoCampus de Gualtar4710‐057BragaPortugal

**Keywords:** Cassava, deterioration, organic acids, polyphenol oxidase, scopoletin, soluble sugars

## Abstract

Food losses can occur during production, postharvest, and processing stages in the supply chain. With the onset of worldwide food shortages, interest in reducing postharvest losses in cassava has been increasing. In this research, the main goal was to evaluate biochemical changes and identify the metabolites involved in the deterioration of cassava roots. We found that high levels of ascorbic acid (AsA), polyphenol oxidase (PPO), dry matter, and proteins are correlated with overall lower rates of deterioration. On the other hand, soluble sugars such as glucose and fructose, as well as organic acids, mainly, succinic acid, seem to be upregulated during storage and may play a role in the deterioration of cassava roots. Cultivar Branco (BRA) was most resilient to postharvest physiological deterioration (PPD), while Oriental (ORI) was the most susceptible. Our findings suggest that PPO, AsA, and proteins may play a distinct role in PPD delay.

## Introduction

Cassava (*Manihot esculenta* Crantz.) is a major tropical root crop grown in Africa, Latin America, Oceania, and Asia, feeding more than 800 million people each day. The root, which is the major edible portion of the plant, is an important source of dietary energy and comprises more than 80% starch (Montagnac et al. 2009; Lyer et al. 2010; Harris and Koomson 2011). Historically, cassava has played an important role in food security as a famine reserve crop. In Eastern and Southern Africa where maize is preferred, but drought is recurrent, cassava, which is, to some extent drought tolerant, is harvested when other crops fail (Rosenthal and Ort 2012). Similarly, cassava provides additional food security when armed conflicts lead to the destruction of above‐ground crops, as it remains viable below ground for up to 36 months (Rosenthal and Ort 2012). While cassava continues to be a vital subsistence crop for small‐scale farmers, it is also an increasingly important crop on both regional and global levels (Rosenthal and Ort 2012). In addition to its role in food security, cassava is being used as a biofuel crop in many countries, including China, Thailand, and Brazil (Dai et al. 2006; Nguyen et al. 2007; Zidenga 2012; Zidenga et al. 2012). Globally, cassava is the fifth most important crop overall in terms of human caloric intake (Rosenthal et al. 2012). However, subsistence and commercial utilization of cassava are affected by its short shelf life that results from a rapid postharvest physiological deterioration (PPD) process, which renders the root unpalatable within 72 h of harvest (Owiti et al. 2011).

Posthaverst physiological deterioration is triggered by mechanical damage, an inevitable result of harvesting operations. PPD then progresses from the site of damage, eventually causing general discoloration of the vascular parenchyma throughout the root. According to previous studies (Sánchez et al. 2013; Uarrota et al. 2014), cassava root deterioration is related to two separate processes: physiological, or primary, deterioration and microbiological, or secondary, deterioration (Acedo and Acedo 2013; Njoku et al. 2014). Physiological deterioration is usually the initial cause of reduced acceptability of roots. It can be observed by the blue‐black streaks in the root vascular tissue that later spread and cause a more general brown discoloration finally leading to unsatisfactory cooking quality and adverse taste (Salcedo et al. 2010; Sayre et al. 2011; Naziri et al. 2014). Primary deterioration also involves changes in oxidative enzyme activities which generate phenols, including catechins and leucoanthocyanidins, which polymerize in later stages to form condensed tannins (Zidenga 2012; García et al. 2013; Sánchez et al. 2013). Microbiological deterioration results from pathogenic rot, fermentation, and/or softening of the roots, and generally occurs when the roots have already become unacceptable because of physiological deterioration.

Few reliable estimates can be found that document the extent of postharvest losses. A systematic assessment of physical losses worldwide by the Food and Agriculture Organization (FAO) suggests that losses of root and tuber crops are in the range of 30% to 60%. In the case of cassava in Africa, losses in 2002 were estimated at 19 million tons out of a total production of 101 million tons across the entire continent (NRI [Link]; Harris et al. 2015). Yet, the magnitude of losses significantly differs across countries and different value chains within a single country as such losses largely depend on how cassava is produced, processed, and consumed, and on the level of coordination among value chain actors (Naziri et al. 2014). Extending the shelf life of cassava by about 2 to 3 weeks would translate into a reduction in financial losses by about $2.9 billion in Nigeria alone over a 20‐year period (Zidenga 2012). The rapid postharvest perishability of freshly harvested cassava roots is a problem not known in any other root and tuber crop. Within 1–3 days of harvest, roots begin to develop an endogenous disorder, typically characterized by blue‐black streaking of the vascular tissues of the xylem, which is accompanied by an unpleasant odor and flavor. PPD profoundly impacts processing as well as marketing of the roots (Lyer et al. 2010). Several approaches have been developed to preserve cassava roots, such as underground storage, storage in boxes with moist sawdust, storage in bags combined with the use of fungicides, pruning plants before harvest, cold storage (2–4°C) for up to 2 weeks, freezing or waxing the roots to prevent access to oxygen, and even chemical treatments (Howeler et al. 2013; Sánchez et al. 2013). However, these methods are too expensive or complicated for handling large volumes of roots and have been restricted mostly to high‐value product chains, such as the consumption of fresh cassava roots (Sánchez et al. 2013). Thus, a major goal of cassava breeding and biotechnology is to increase its shelf life by delaying the onset of PPD. Such efforts would expand the industrial applications of cassava worldwide (Zidenga 2012).

Molecular and biochemical studies of PPD have pointed to reactive oxygen species (ROS) production as one of the earliest events in the process, and many other compounds have been reported (Buschmann et al. 2000; Reilly et al. 2003). Specific genes involved in PPD have been identified and characterized, and their expression has been evaluated (Reilly et al. 2007; Timothy 2009). Several secondary metabolites, particularly hydroxycoumarins, accumulate in the process (Bayoumi et al. 2008; Bayoumi et al. 2010). However, more research is necessary to better understand the biochemical changes involved in PPD of cassava roots. Accordingly, this study aimed to evaluate the biochemical changes involved in PPD in four cassava cultivars, including fresh roots (hereinafter designated as nonstored samples) and root samples stored up to 11 days. Using metabolomic techniques integrated with chemometric tools, we further assessed biochemical markers of PPD. Supervised and unsupervised methods of data analysis were also used to discriminate among cassava samples during postharvest physiological deterioration.

## Material and Methods

### Selection of cassava cultivars

Cassava cultivars were provided by the Santa Catarina State Agricultural Research and Rural Extension Agency (EPAGRI), specifically, the experimental station of Urussanga, and were produced over the 2011/2012 growing season. Four cultivars were selected for this study as follows: SCS 253 Sangão (hereinafter designated as SAN), Branco (hereinafter designated as BRA, a landrace), IAC576‐70 (hereinafter designated as IAC, a commercial variety), and Oriental (hereinafter designated as ORI, a landrace). The cultivars were selected as they are widely used by small farmers and lacking research efforts.

### Plant materials and postharvest physiological deterioration

On‐farm trials were carried out at the Ressacada Experimental Farm (Plant Science Center, Federal University of Santa Catarina, Florianópolis, SC, Brazil – 27°35′48″ S, 48°32′57″W) in September 2011, using the four EPAGRI cassava cultivars noted above. The experimental design was in complete randomized blocks, with 4 blocks (6.3 × 15 m^2^/block) spaced at 1 m. Each block consisted of four plots (12 × 1.2 m^2^/plot), spaced at 0.5 m. Cassava stakes of length 15 cm were planted upright and spaced 1 × 1 m. Each plot was considered an experimental unit to which a treatment was applied, and all land operations were mechanized. Soil fertility had already been determined by chemical analysis, and cultivation was performed manually.

Cassava root samples were harvested from 12‐month‐old plants for analysis. Immediately after harvest, the roots were washed with sterilized water, and both proximal and distal parts of the root were removed. Cross sections were made (0.5‐1 cm) over the remaining root and stored at room temperature (66‐76% humidity, ±25°C). Induction of PPD was performed during 11 days using different roots in the same batch. Monitoring the development of PPD and associated metabolic disturbances was performed daily after induction of PPD. Fresh samples and those at 3, 5, 8, and 11 days postharvest were collected at each point, dried (35‐40°C/48 h) in an oven, milled with a coffee grinder (Model DGC‐20N series, Cadence, Brazil), and kept for analysis. For enzymatic analysis, nonstored samples were collected and stored (‐80°C) until analysis. PPD was also induced using two other methodologies, including storage of the entire root and the method of Wheatley (1982) whereby only the proximal and distal parts of the roots were removed without slicing the remaining part. The experiment was conducted using the same room conditions as to the method described firstly.

### Postharvest Physiological Deterioration Scoring

Five independent evaluations of PPD were carried out. For each harvest, a random sample of three sliced roots from each plant variety was scored according to visual observations of sliced cassava roots (from 1 –10% of deterioration to 10–100% of deterioration) at each stage of PPD (i.e., 3, 5, 8, and 11 days postharvest) and imaged with a digital camera (OLYMPUS FE‐4020, 14 megapixel, China). The mean PPD score for each root was calculated by averaging the scores for the three transversal sections and five evaluations (see Table S1). Roots showing symptoms of microbial rotting, which would not be reflective of PPD, or those showing inset activity were discarded.

### Dry matter content (%)

To obtain the dry matter content of cassava samples, 10–30 g of chopped and grated fresh roots were weighed, and oven dried at 60°C for 48 h. Dry matter was expressed as the percentage of dry weight relative to fresh weight (Morante et al. 2010).

### Polyphenol oxidase activity during PPD

For polyphenol oxidase (PPO) analysis, 2 g of fresh tissue were homogenized with 0.6 g of PVPP and 8 mL of 50 mmol/L (pH 7) phosphate buffer, followed by recovery of the supernatant by filtration and centrifugation (3220g, 4°C, 15 min, 18 cm of rotor radius). The product constituted an enzymatic extract. PPO activity was measured using 2.85 mL of 0.2 mmol/L (pH 7) phosphate buffer, 50 *μ*L of catechol (60 mmol/L) as substrate, and 100 *μ*L of enzymatic extract at 25°C. Changes in absorbance (420 nm) were recorded over a 5‐min period in a UV‐vis spectrophotometer (Spectrumlab D180, BEL Photonics, Brazil; Montgomery and Sgarbieri 1975). Activity was expressed as units of activity (UA), and one unit of PPO was defined as the change in one unit of absorbance per second.

### Ascorbic acid determination during PPD

Ascorbic acid (AsA) content was assayed as described previously with slight modifications (Omaye et al. 1979). The extract was prepared by grinding 1 g of sample with 5 mL of 10% TCA, centrifuged (2465g, 18 cm rotor radius, 20 min), and then re‐extracted twice. To the supernatant, 1.0 mL of extract and 1 mL of DTC reagent (2,4‐dinitrophenylhydrazine–thiourea–CuSO_4_) were added to a total volume of 10 mL and incubated (37°C, 3 h), followed by the addition of 0.75 mL ice‐cold 65% H_2_SO_4_ (v v^−1^). The mixture was allowed to stand for 30 min at 30°C. The resulting color was read at 520 nm in the spectrophotometer (Spectrumlab D180, BEL Photonics, Brazil). A standard AsA curve was constructed to determine content (*y* = 0.0361*x*,* r* ^2 ^= 0.99, 0 to 1000 mg mL^−1^) and the results were expressed in *μ*g g^−1^ (ppm) of fresh weight.

### Protein extraction and quantification from cassava roots during PPD

At each sampling time, root tissues were grated using a food processor (Walita‐Master Plus, Brazil) and stored at −80°C before use. The frozen tissue was ground under liquid nitrogen to a fine powder using a prechilled pestle and mortar. Then, 5 g of tissue were added to a prechilled 50 mL tube containing 20 mL of extraction buffer (phosphate buffer 0.1 mol/L, pH 6.4, 0.25 g of PVP), 200 *μ*L of 1 mmol/L DTT, and 200 *μ*L of 1 mmol/L EDTA. Tubes were vortexed vigorously and transferred to a horizontal shaker (300 rpm) for 1 h. Tubes were centrifuged (5000 rpm, 20 min), and the supernatant was recovered by filtration, transferred to a fresh tube, and stored at −20°C (Reilly 2001).

The protein content of each sample was determined using the Bradford protocol (Bradford 1976) with small modifications. A calibration curve was constructed using bovine serum albumin (BSA) (Sigma‐Aldrich, St. Louis, MO) as standard. Protein solutions were prepared in 0.15 M NaCl, and a series of dilutions was prepared (0 to 100 mg mL^−1^, *y* = 0.0082*x*,* r* ^2 ^= 0.96) to build the standard curve.

### Extraction and quantification of soluble sugars and organic acids by HPLC during PPD

Nonstored samples and those at 3, 5, 8, and 11 days postharvest were collected. The outer and inner bark were removed and crushed with a food processor, as previously described, and dried in an oven at 35°C (48 h). After oven drying, samples were again crushed with a coffee grinder to obtain a fine powder, sieved and stored at room temperature for analysis.

Sugars and organic acids were extracted from 0.5 g of cassava root flour samples in 10 mL of mobile phase (H_2_SO_4_, 5 mmol/L) and determined accordingly (Chinnici et al. 2005). Briefly, the suspension was homogenized using an Ika Works Ultra‐Turrax (Ika, China) Digital Homogenizer and mixed slowly using a horizontal shaker (Microplate shaker, 330 rpm) for 30 min. The suspension was centrifuged (12879g, 10 min) and filtered through a 0.22 *μ*m disposable syringe membrane filter, followed by collection of the supernatant. Sugars and organic acids were analyzed by HPLC using a Bio‐Rad Aminex HPX‐87H HPLC column equipped with a UV detector (MWDG 1365D for organic acids), connected in series with a refractive index detector (RID G 1362A for sugars) and an injection valve fitted with a 15 *μ*L loop. The samples were separated isocratically at 0.6 mL min^−1^ at 30°C.

Retention times and standard curves were prepared for the following sugars and organic acids (see Table 1). Three consecutive injections (10 *μ*L) were performed. Sugars and organic acids were expressed (mg g^−1^) as mean ± standard deviation.

**Table 1:** The HPLC standard curves prepared for sugars and organic acids studied. Three consecutive injections (10 *μ*L) were performed. Sugars and organic acids were expressed (mg g^−1^) as mean ± standard deviation

Group of compound	Name of the compound^1^	Code	Standard curve	*r* ^2^
Soluble Sugars	Glucose	G7528	*y* = 26748656*x*‐1523663	0.99
	Fructose	F2543	*y* = 26028204*x*‐8253663	0.99
	Raffinose	R0514	*y* = 22680182*x* + 45255.3	0.99
	Sucrose	S7903	*y* = 22582989*x* + 727997.7	0.99
	Citric	CO759	*y* = 3281.1*x* + 46046	0.99
Organic acids	Malic	240179	*y* = 2498.2*x* + 3816.4	0.99
	Succinic	S3674	*y* = 1737.8*x*−4255.3	0.99
	Fumaric	R412205	*y* = 4047.85*x*−5748.3	0.99

All reagents were acquired from Sigma‐Aldrich

### Scopoletin extraction and quantification during PPD

Cassava root flour samples (1 g) were placed in 50 mL falcon tubes containing 2 mL 98% ethanol and homogenized with an Ika Works Ultra‐Turrax T18 ( IKA, China) for 30 sec. The suspension was vortexed (1 min), incubated (microplate shaker, 600 rpm, 30 min), and centrifuged (9861, 5 min). The extract was filtered on a Whatman # 1 paper and through a 0.22 *μ*m nylon membrane. Samples were transferred to 1.5 mL vials for HPLC (Agilent 1200 series, Agilent Technologies, Waldbronn, Germany) analysis (Buschmann et al. 2000). To accomplish this, samples (50 *μ*L) were injected into the Agilent 1200 series HPLC equipped with a reverse‐phase column (Techsphere BDS C18, 250 mm × 4.6 mm, 5 *μ*m) and a diode array detector. The column was kept at 25°C, and acetonitrile and 0.5% phosphoric acid (v v^−1^) in aqueous solution were used as mobile phase. The gradient profile was 60 – 1% for 30 min with a 0.5 mL min^−1^ flow and 50 *μ*L injection volume. Scopoletin was detected at 215, 280, and 350 nm according to its retention time with a standard compound sample (Sigma–Aldrich: scopoletin ≥ 99% – No. S2500). Scopoletin quantification was determined through a calibration standard curve (y = 158159.59x, *r* ^2 ^= 0.9993, 1–75 mg l^−1^). Three consecutive injections (10 *μ*L) were performed. Quantifications were made on a dry weight basis, and data were represented in nmol g^−1^, as mean ± standard deviation.

### Statistical analysis

Each harvest was considered independent from the others. Roots of the same cultivar in the four field blocks were combined into one bulk volume, and repetitions (*n* = 3) were made according to the bulk sample. All statistical analyses were carried out using R software (R core team‐2015, version 3.1.1), using their respective packages and scripts. All values were presented as mean ± standard deviation of three repetitions (*n* = 3). Two‐way ANOVA and multivariate analysis were applied when necessary.

## Results and Discussion

### PPD scoring

**Figure 1:**
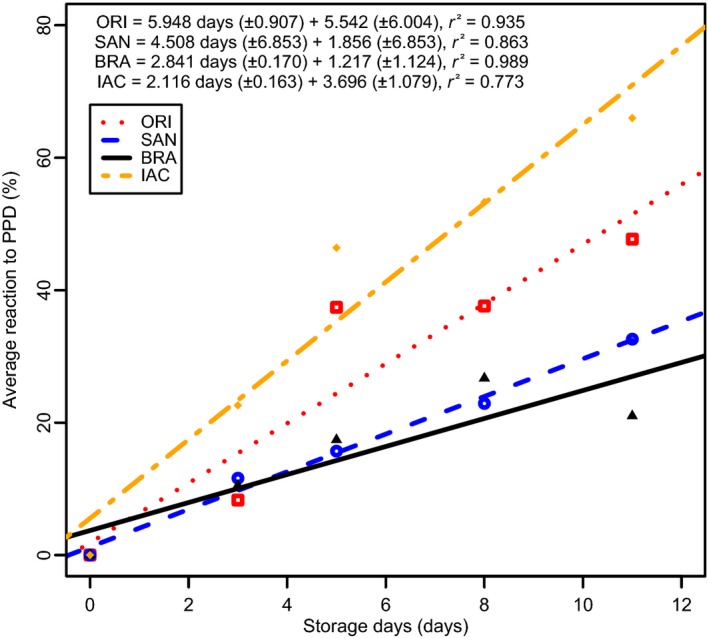
Average reaction to PPD (grouping together the three methods for assessing PPD) through time for the four cultivars involved in this study. The result of the linear regression analysis is also provided. Standard errors of the parameters in the regression analyses are given in parentheses.

Results of PPD scoring of the four genotypes studied showed that ORI was the most susceptible cultivar to PPD, followed by SAN, while BRA and IAC were found to be more tolerant (Table 2). BRA and IAC showed a slower deterioration rate when compared with the faster deterioration rates observed at ORI and SAN. Similar regression coefficients were found among tolerant clones (0.935, 0.863 for ORI and SAN, respectively). Figure 1 shows regression models for the four cultivars during storage. For all PPD methods, an increasing rate of deterioration throughout storage was observed. A clear relationship was found when using the average value of the three methods as shown in Figure 1. Cassava roots deteriorated faster after they were sliced. The root slicing method was applied to all analyses in this study. The entire root method was found to preserve the best postharvest quality, and BRA was the most resilient based on all testing methods.

**Table 2:** Comparison of methods of PPD induction in four cultivars studied. Values are represented as mean scores of five independent evaluations in percentage (%), from zero to 100% of deterioration during different storage times

Cultivar	Method of PPD	3 days	5 days	8 days	11 days
SANGÃO	Root slicing	15.70	70.70	89.30	100.00
	Wheatley	9.30	5.70	8.60	17.10
	Entire root	0.00	35.70	15.00	26.00
Average		8.3	37.4	37.6	47.7
BRANCO	Root slicing	34.90	46.40	68.60	81.50
	Wheatley	0.00	0.70	0.00	15.00
	Entire root	0.00	0.00	0.00	1.40
Average		11.6	15.7	22.9	32.6
IAC576‐70	Root slicing	31.50	49.30	67.20	91.00
	Wheatley	0.00	2.90	1.40	5.00
	Entire root	0.00	0.00	11.40	3.60
Average		10.5	17.4	26.7	21.0
ORIENTAL	Root slicing	59.90	87.10	100.00	100.00
	Wheatley	7.90	17.20	24.30	33.60
	Entire root	0.00	35.00	35.70	64.30
Average		22.6	46.4	53.3	66.0

### Polyphenol oxidase

**Figure 2:**
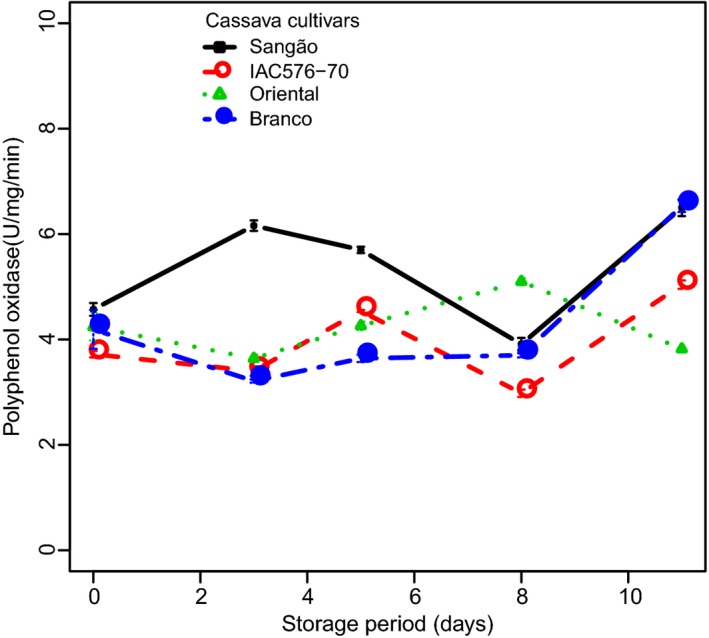
Changes in the activity of polyphenol oxidase in cassava cultivars during PPD. Each data point is presented as mean ± standard deviation (*n* = 3) in units per milligram*minutes (U mg^−1^ min^−1^).

Polyphenol oxidase results during storage are summarized in Figure 2. By comparing the mean PPO values from each cultivar, we found that BRA and ORI did not differ significantly (*P* < 0.05). During storage, a small decrease in PPO activity at day 8 was observed for IAC and SAN; however, in general, PPO activity varied similarly in the tolerant (BRA/IAC) and susceptible (SAN/ORI) cultivars. PPO activity based on analysis of nonstored samples and those at day 3 of PPD, showed significant difference from samples at days 5, 8, and 11 (*P* < 0.05). SAN showed higher PPO activity than other cultivars, particularly at days 3 and 5, most likely related to the higher PPD scores for this cultivar.

When PPO values were correlated with PPD (see Fig. S1A), we found a high negative correlation and a clear discrimination between tolerant cultivar (BRA/IAC) and susceptible (ORI/SAN) ones. A similar trend in correlation values could be observed (0.790, 0.785, and 0.970, 0.93, respectively), meaning that higher activity of PPO in cassava roots was correlated with the reduced deterioration in that cultivars. Fluctuations in PPO, as shown in Figure 2, could be attributed to differences in genotype, as well as pre‐ and postharvest handling conditions.

PPO has been identified as a major cause of darkening in raw Asian noodles and other wheat products (Anderson et al. 2006), as well as browning induced in mechanically damaged potatoes (Batistuti and Lourenço 1985). PPO catalyzes the oxidation of phenols into quinones, which subsequently polymerize into brown pigments, a phenomenon that has also been reported in avocado (Gomez‐Lopez 2002) and browning in marula fruits (Mdluli 2005).

### Ascorbic acid

**Figure 3:**
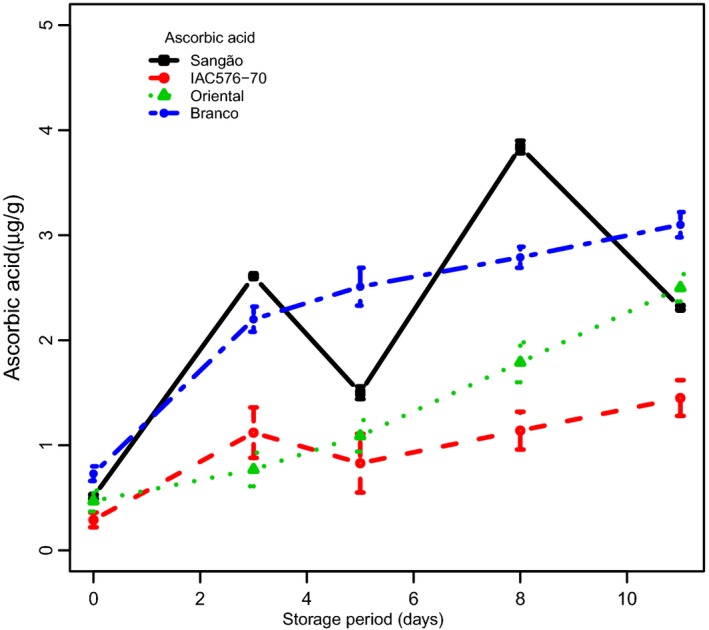
Changes in the concentrations of ascorbic acid in root samples of cassava cultivars during PPD. Each data point is presented as mean ± standard deviation (*n* = 3) in (*μ*g g^−1^).

Our results showed that AsA gradually accumulated over time in all cultivars studied (Fig. 3). Interestingly, the cultivars with tolerance to PPD, IAC, and BRA, demonstrated the most extreme contrast for AsA, while the two susceptible cultivars, SAN and ORI, after several days of evaluation, showed only intermediate values of AsA. ORI showed a sharp increase in AsA after the third day of storage. SAN, on the other hand, showed a strong fluctuation in AsA activity through time, and, as a result, its behavior is difficult to define.

Two‐way ANOVA showed differences in AsA between BRA/SAN and ORI/IAC. During storage, nonstored samples were statistically different from samples at days 3, 5, 8, and 11, respectively (*P* < 0.05). Although Figure 3 shows increases in AsA during storage until day 3, followed by fluctuation on other days, a clear correlation was found when assessing the levels of AsA between tolerant and susceptible cultivars in the context of PPD. Specifically, tolerant cultivars (BRA/IAC) behaved similarly and presented low negative correlations (0.785 and 0.793, respectively) when compared with susceptible cultivars (ORI/SAN) (0.969 and 0.932, respectively) (see Fig. S1B). In susceptible cultivars, the levels of AsA appeared to impact the degree of deterioration. Thus, the high levels of AsA presented by the tolerant cultivar (BRA) may be interpreted as causing a delay in PPD.

A wide range of factors, such as genotype, as well as pre‐ and postharvest conditions, may influence the AsA content. Losses of AsA during storage have been reported previously in many fruits, depending on storage conditions (Kabasakalis et al. 2000). Moreover, AsA has been reported to act as an antioxidant, thus prolonging the shelf life of commercial products (Fung and Luk 1985). In addition, lower storage temperatures have been reported to reduce the loss of AsA and the incidence of storage disorders in peas, broccoli, and spinach (Felicetti and Mattheis 2010). Although many studies have reported on the effects of AsA in many different crops, results of this study indicate that high levels of AsA may have a positive effect on PPD in cassava roots, thus inviting more research to better understand the precise role of AsA.

### Changes in total proteins and dry matter content

**Figure 4:**
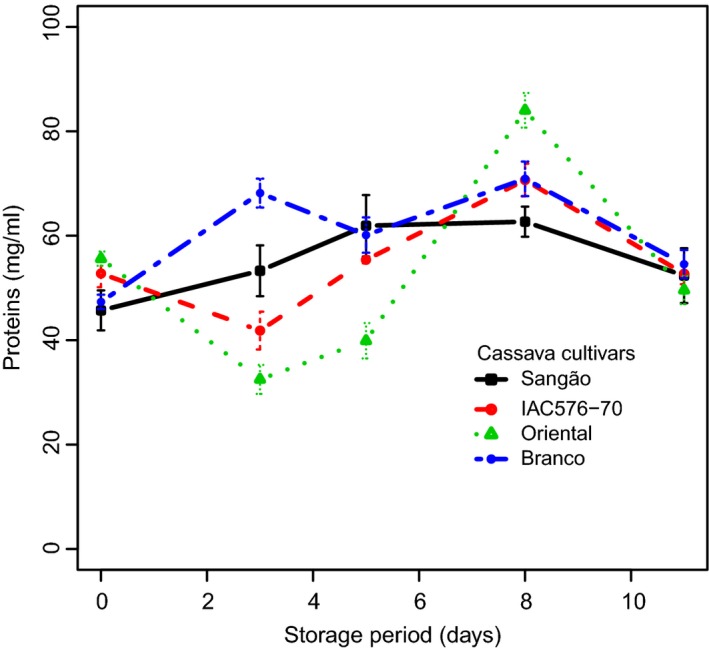
Changes in the concentrations of total proteins in roots of cassava cultivars during storage. Each data point is presented as mean ± standard deviation (*n* = 3) in (mg mL^−1^).

The results of total protein contents in the roots of the sampled cultivars are summarized in Figure 4. Protein amounts significantly changed among the cultivars over the experimental period, except for days 5 and 11 of storage, revealing a genotype‐specific behavior for that variable. Interestingly, while a considerable increase in protein amount was observed on day 3 in BRA, which could possibly be attributed to its tolerance to PPD, IAC showed contrasting behavior. This variable also differed between BRA and ORI, and correlations between PPD and protein levels are all summarized in Figure S1C. Lower negative correlations and similar trends in deterioration can be observed in all cultivars with protein levels, but such results make it difficult to define trends.

Dry matter content was also determined in nonstored samples and was correlated with PPD at days 3, 5, 8, and 11. A positive correlation was established between dry matter and PPD, leading to the implication that cultivars with a high level of dry matter are more prone to suffer from PPD (see Fig. S1D). It was also observed that these correlations decreased as PPD progressed; thus, at days 3, 5, 8, and 11, we found correlation values of 37%, 25%, 17%, and 11%, respectively, and these findings confirm previous studies (Ceballos et al. 2012; Sánchez et al. 2013). Taken together, it can be concluded that cassava roots with lower content of dry matter have longer shelf life.

### Scopoletin content during PPD

The HPLC results of scopoletin contents during PPD are summarized in Table 3 and a representative chromatographic profile for the studied cultivars during PPD is provided in Figure S2. It was found that tolerant cultivars presented high levels of scopoletin at the starting point (nonstored samples) and at day 11 of PPD. A different trend was observed for susceptible cultivars, which presented low levels of scopoletin in nonstored samples and at day 11 of PPD. Fluctuations during PPD can be attributed to such factors as genotype and PPD conditions. By correlating scopoletin with PPD (Figure S3), we found that those cultivars with low levels of scopoletin presented a high degree of deterioration (ORI/SAN) when compared with tolerant ones (BRA/IAC). Unlike other variables, the positive correlation between scopoletin and PPD led to a clear separation of tolerant and susceptible cultivars. Two‐way ANOVA showed significant differences (*P* < 0.05) in scopoletin levels in all cultivars and during storage. BRA showed a high level of scopoletin when compared to ORI.

**Table 3:** HPLC analysis of scopoletin (mmol g^−1^) during PPD in cassava root tubers of the four cassava cultivars studied. Data are represented as mean ± standard deviation of two repetitions (*n* = 3). Letters in the column represent significant differences (Tukey HSD test, *P* < 0.05)

PPD days	BRA	ORI	SAN	IAC
0	91.46c	18.59b	25.99d	64.26e
3	92.11c	124.89a	45.40c	81.81d
5	95.00bc	48.17b	120.80a	123.90b
8	193.96ab	81.79ab	125.81a	214.00a
11	223.08a	54.94b	66.65b	98.10c

Values are represented as mean of three repetitions (*n* = 3) in mmol g^−1^ of dry weight. Different letters in the column represent significant statistical differences (Tukey HSD, *P* < 0.05).

PPD has been described as a physiological process that results from altered gene expression (Reilly et al. 2003; Reilly et al. 2004) and the accumulation of secondary metabolites. Among these secondary metabolites are found hydroxycoumarins (e.g., scopoletin) which show antioxidant properties, and by oxidation and polymerization, they confer the typical blue/black phenotype to root cassavas undergoing PPD. Hydroxycoumarins are important in plant defense as phytoalexins by the induction of biosynthesis following various stress events, such as wounding or bacterial and fungal infections. Additionally, they display a wide range of pharmacological activities, including anticoagulation (Mueller 2004), anti‐inflammatory (Silvan et al. 1996), antimicrobial (Smyth et al. 2009), and antitumoral (Grazul and Budzisza 2009). However, while their biosynthesis pathway in cassava has not been elucidated (Wheatley 1982; Bayoumi et al. 2010), their accumulation in the biomass of that species during root deterioration has been previously reported (Wheatley 1982; Wheatley and Schwabe 1985; Sánchez et al. 2013). An uptake of scopoletin regulated by interaction among plant hormones, such as salicylic acid, was also reported (Taguchi et al. 2001).

### Soluble sugar content during PPD

**Figure 5:**
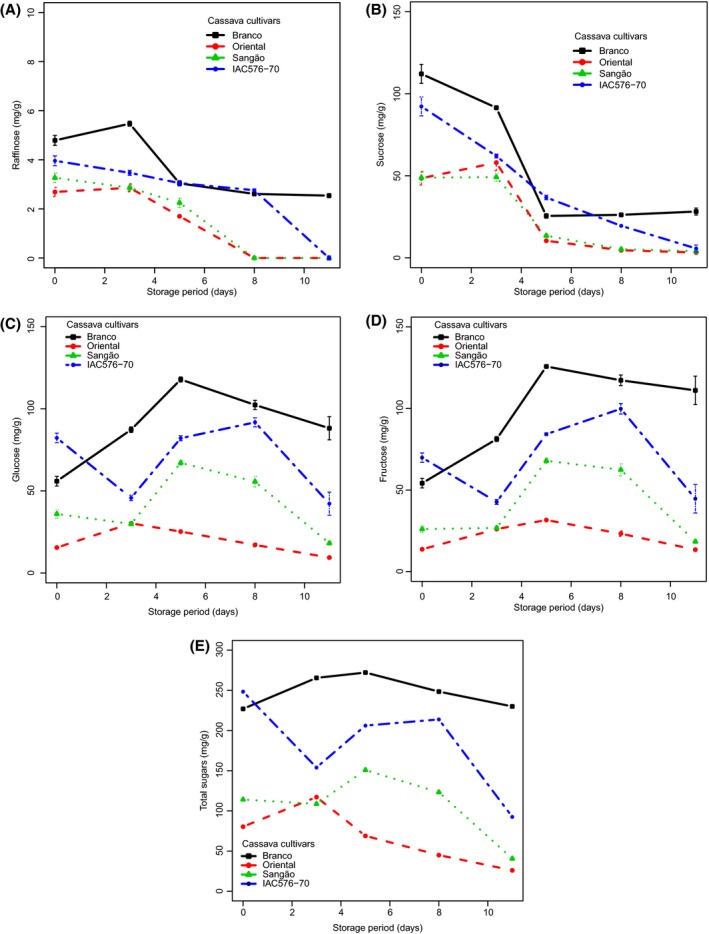
Changes in the concentrations of soluble sugars in cassava cultivars during storage. Each data point is presented as mean ± standard deviation (*n* = 3) in (mg g^−1^). (A) Raffinose, (B) Sucrose, (C) Glucose, (D) Fructose, and (E) Total sugars.

Soluble sugar contents detected in cassava roots during PPD are summarized in Figure 5A–E and Table S2. Figure S4 shows a typical chromatogram of soluble sugars detected in BRA during PPD, and Figure 5A–E shows changes in soluble sugars, including raffinose, sucrose, glucose, fructose, and total sugars, during storage. Significant differences (*P* < 0.05) were found in soluble sugar amounts during storage for each cultivar. Raffinose content was observed to decrease in all cultivars, except BRA, which showed a small increase up to the third day of storage (Fig. 5A). Decrease in sucrose was also observed, except for ORI where increases were observed until day 3 of storage (Fig. 5B). Glucose, fructose, and total sugar content showed similar trends. A small decrease (SAN, IAC), followed, in turn, by increases, was observed in all cultivars studied (Fig. 5C–E). Glucose and fructose were the main sugars found in all samples studied. Researchers working with susceptible and tolerant cultivars of cassava stored for 14 days at ambient conditions also reported similar results for soluble sugars (Sánchez et al. 2013).

### Organic acids during PPD

**Figure 6:**
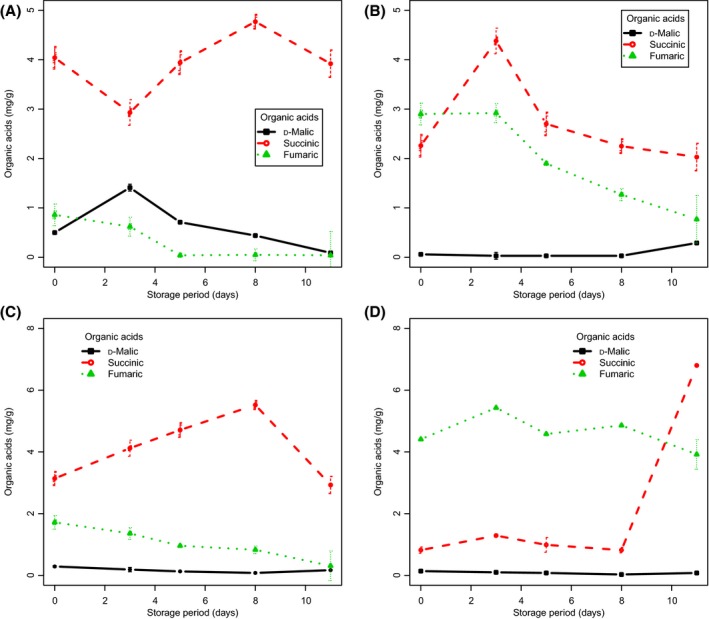
Changes in the concentration of organic acids in roots of cassava cultivars during PPD. Each data point is presented as mean ± standard deviation (*n* = 3) in (mg g^−1^). (A) Branco, (B) Oriental, (C) Sangão, and (D) IAC 576‐70.

Figure 6A–D summarizes the results of the organic acid analysis performed. Changes in the contents of these metabolites for the studied cultivars can also be reviewed in Figure S5 and Table 3. The organic acid profiles of cassava roots sampled significantly differed. The main organic acids predominantly found during PPD were succinic and fumaric acids. In PPD‐tolerant BRA, succinic acid and malic acid were the major compounds detected (Fig. 6A). Small decreases, followed by an increase in succinic acid, were observed in those samples. In ORI (Fig. 6B), increases in succinic acid and decreases in fumaric acid during PPD were also observed, while the level of malic acid remained quite constant during PPD. In SAN (Fig. 6C), increases in succinic acid up to day 8 of PPD were found; meanwhile, the levels of fumaric and malic acids decreased. For IAC, no trend was detected for succinic and fumaric acids (Fig. 6D). The chromatographic profile (Fig. S3) of BRA samples (nonstored) at days 3 and 5 of PPD shows other organic acids detected in small amounts, for example, phytic acid (data not shown). In tolerant clones (BRA/IAC), we found succinic acid to be the main acid related to PPD; in susceptible cultivars (ORI/SAN), we found that fumaric acid was the main acid related to PPD.

As primary metabolic products, organic acids play a regulatory role in plant growth and development. Organic acids are metabolically active solutes in cellular osmoregulation and surplus cationic balance, acting as key components in response to nutritional deficiencies, metal ion accumulation, and plant–microorganism interaction. Organic acids can also enhance resistance to diseases and inhibit oxidation during storage at low temperature, resulting in a significant extension of storage life for plant biomasses (Sun et al. 2012). They have also been related to maintenance of membrane integrity in stress conditions (Gunes et al. 2007). Since the postharvest physiological deterioration properties of stored cassava remain largely unknown, studying the metabolic profile of organic acids in postharvest stored cassava roots can lead to a better understanding of PPD.

### Multivariate statistical analyses

Chemometric techniques that include multivariate models (e.g., principal component analysis (PCA), hierarchical cluster analysis (HCA), partial least squares discriminant analysis (PLS‐DA), linear discriminant analysis (LDA), and support vector machines (SVM)) can be applied to complex and collinear data to extract relevant information. Both nonsupervised (HCA, PCA) and supervised (PLS‐DA, LDA, and SVM) methods reduce large datasets by combining collinear variables into a small number of latent variables (LVs), which are then used in place of the full dataset to build prediction models (Sills and Gossett 2012; Tang et al. 2014).

**Figure 7:**
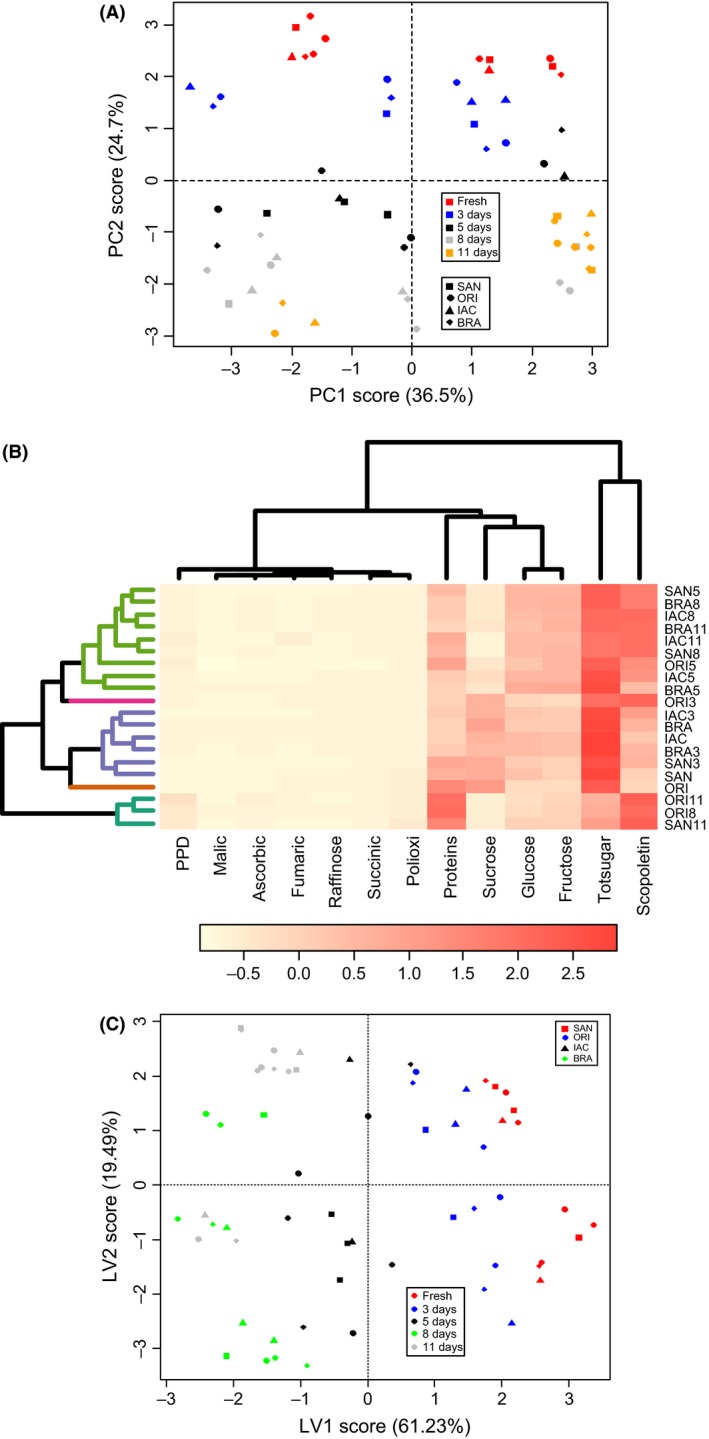
(A) Scores plot of a two‐component PCA model from the metabolic dataset of cassava roots showing sample clustering according to metabolic fingerprinting and the percentage of variance captured by each PC. (B) A seriated cluster heat map (HCA), with cophenetic correlation coefficient of 78.1%. (C) PLS‐DA components score plot of cassava samples during PPD, taking into consideration all the metabolites analyzed. PLS‐1 (*x*‐variate 1) = 61.23%; PLS‐2 (*x*‐variate2) = 19.43% of variance explained.

When PCA was applied to the scaled data in the present work, a clear separation was noted between nonstored samples and those that had undergone 3 days of PPD. A clear separation between tolerant and susceptible cultivars was found (Fig. 7A). The total variance explained by the two principal components was 63.7%, that is, PC1 (37%) and PC2 (26.7%). The loadings plot showed that samples grouped in PC1+/PC2+ according to their values of polyphenol oxidase; in PC2+/PC1− according to fructose, scopoletin, proteins, and ascorbic acid contents; in PC1‐/PC2− according to glucose, raffinose, total sugars, and organic acids (malic and succinic acid). Samples grouped in PC1+/PC2− showed similarity in their fumaric acid concentrations. The clustering method was able to separate nonstored (fresh) samples with those at 3 days of storage. Other samples were clustered together on days 5 and 8 of storage based on similar behavior.

When a serrated cluster heat map was applied to the data (Fig. 7B), results similar to those found in PCA were detected, and four major clusters were detected to occur: group 1 (SAN, ORI, SAN3, and ORI5); group 2 (IAC11, ORI8, ORI11, and SAN8); group 3 (BRA, IAC, ORI3, and BRA3), and group 4 (SAN8, BRA11, BRA5, SAN5, IAC5, BRA8, and IAC8). A cophenetic correlation coefficient of 78.1% was found. The cluster heat map reveals major metabolic components that influenced the clustering noticed. Proteins and ascorbic acid were the major compounds related to group 1, polyphenol oxidase activity for group 2, glucose, succinic acid, and total sugars contents for group 3, and sucrose, raffinose, and malic acid for the last group.

Using supervised methods, for example, PLS‐DA (Fig. 7C), a better separation was found (accuracy of 88.4%), when compared to PCA. BRA grouped with IAC, and ORI grouped with SAN. Most nonstored samples and those at day 3 of storage were found in the same component (x‐variate), and those at day 5, 8, and 11 grouped in the same component as their presented similarities. Again, the built model was capable of predicting and separating tolerant and susceptible cultivars. The total variances explained from the axis were 80.72%, that is, 61.23% from latent variable 1 (*x*‐variate 1) and 19.49% from latent variable 2 (*x*‐variate 2). The loading values showed that samples grouped in x‐variate 1+ according to malic and fumaric acids, raffinose, sucrose, and total sugars, while for the *x*‐variate 1‐, samples grouped according to the values of glucose, fructose, scopoletin, ascorbic acid, proteins, polyphenol oxidase, and degree of PPD. Samples were also grouped in *x*‐variate 2+ by similar amounts of malic and fumaric acids, ascorbic acid, and polyphenol oxidase, while in x‐variate 2‐, samples grouped according to the amounts of sugars, succinic acid, scopoletin, and proteins.

## Conclusions

Based on the biochemical data herein presented, metabolic differences in cassava root samples could be correlated with deterioration state and cultivar. Our findings indicate that polyphenol oxidase, ascorbic acid, and proteins are all upregulated in the initial stages of PPD, up to 72 h, and, as such, may related to PPD. Scopoletin biosynthesis is increased at the beginning of the PPD process, but the underlying mechanism remains to be elucidated. Fumaric and succinic acids were the main organic acids found in tolerant (BRA/IAC) and susceptible (ORI/SAN) clones. Our study suggests that PPO, AsA, and proteins may all play a role in PPD delay. We also found that the entire root method worked best for maintaining the postharvest quality as determined by observing samples from the most resilient cultivar to PPD, BRA, and the most susceptible, ORI. Finally, the pattern recognition models, both supervised and unsupervised, classified samples according to their metabolic profiles and degree of deterioration. Cassava roots with lower dry matter have longer shelf life.

## Conflict of Interest

Authors declare no conflicts of interest.

## Supporting information


**Figure S1.** Correlations between PPD with Polyphenol oxidase (A), with Ascorbic acid (B),proteins (C), dry matter (D).
**Figure S2.** Chromatographic profiles (HPLC, detection wavelength at 350 nm) of cassava root extracts (cultivar Branco) showing the peaks of the identified hydroxycoumarins, the major peak being scopoletin.
**Figure S3.** Correlations between PPD and scopoletin. Linear regression correlations are also provided in the figures.
**Figure S4.** Representative chromatograms (HPLC, 350 nm) of cassava root extracts (cultivar Branco) showing the major peaks of the soluble sugars identified (glucose, sucrose, and fructose).
**Figure S5.** Chromatographic profile (HPLC, 350 nm) of cassava root extracts (cultivar Branco) showing the peaks of the organic acids detected.
**Table S1.** Attributes of PPD scoring of cassava root samples (from 1–10% of root deterioration to 10–100% of deterioration) based on visual observation of root slices at different days of storage (3, 5, 8 and 11).
**Table S2.** HPLC analysis of soluble sugars (mg g^−1^) during PPD in cassava root tubers of the four cassava cultivars studied.
**Table S3.** HPLC analysis of organic acid contents (mg g^−1^) during PPD in cassava root tubers of the four cassava cultivars studied.
