# Supplementary material for: Toward better understanding of postharvest deterioration: biochemical changes in stored cassava (Manihot esculenta Crantz) roots:
Source: Food Sci Nutr. 2015 Oct 26;4(3):409–22. doi: 10.1002/fsn3.303 (PMC4867761; doi:10.1002/fsn3.303)
Supplement: Supplementary file 1 — Figure S1. Correlations between PPD with Polyphenol oxidase (A), with Ascorbic acid (B),proteins (C), dry matter (D). Figure S2. Chromatographic profiles (HPLC, detection wavelength at 350 nm) of cassava root extracts (cultivar Branco) showing the peaks of the identified hydroxycoumarins, the major peak being scopoletin. Figure S3. Correlations between PPD and scopoletin. Linear regression correlations are also provided in the figures. Figure S4. Representative chromatograms (HPLC, 350 nm) of cassava root extracts (cultivar Branco) showing the major peaks of the soluble sugars identified (glucose, sucrose, and fructose). Figure S5. Chromatographic profile (HPLC, 350 nm) of cassava root extracts (cultivar Branco) showing the peaks of the organic acids detected. Table S1. Attributes of PPD scoring of cassava root samples (from 1–10% of root deterioration to 10–100% of deterioration) based on visual observation of root slices at different days of storage (3, 5, 8 and 11). Table S2. HPLC analysis of soluble sugars (mg g−1) during PPD in cassava root tubers of the four cassava cultivars studied. Table S3. HPLC analysis of organic acid contents (mg g−1) during PPD in cassava root tubers of the four cassava cultivars studied. [file FSN3-4-409-s001.doc]

**Appendix A: Supplementary data**

**Captions for supplementary data**

Figure 1. Correlations between PPD with Polyphenol oxidase (A), with Ascorbic acid (B),proteins (C), dry matter (D). Linear regression correlations are also provided in the figures.

Figure 2. Chromatographic profiles (HPLC, detection wavelength at 350 nm) of cassava root extracts (cultivar Branco) showing the peaks of the identified hydroxycoumarins, the major peak being scopoletin.

Figure 3. Correlations between PPD with scopoletin. Linear regression correlations are also provided in the figures.

Figure 4. Representative chromatograms (HPLC, 350 nm) of cassava root extracts (cultivar Branco) showing the major peaks of the soluble sugars identified (glucose, sucrose, and fructose).

Figure 5. Chromatographic profile (HPLC, 350 nm) of cassava root extracts (cultivar Branco) showing the peaks of the organic acids detected.

Table 1. Attributes of PPD scoring of cassava root samples (from 1-10% of root deterioration to 10-100% of deterioration) based on visual observation of root slices at different days of storage (3, 5, 8 and 11). Values represent the mean and standard deviations of five independent evaluations (harvests).

Table 2. HPLC analysis of soluble sugars (mg g^-1^) during PPD in cassava root tubers of the four cassava cultivars studied. Data are represented as mean ± standard deviation of two repetitions (n=3). Letters in the column represent significant differences (TukeyHSD test, p<0.05). Numbers after cultivar name means days of PPD (e.g., BRA3 means sample of BRA cultivar with 3 days of PPD). Statistical analyses were made separately for each cultivar and each compound found.

Table 3. HPLC analysis of organic acid contents (mg g^-1^) during PPD in cassava root tubers of the four cassava cultivars studied. Data are represented as mean ± standard deviation of two repetitions (n=3). Letters in the column represent significant differences (TukeyHSD test, p<0.05). Numbers after cultivar name mean days of PPD (e.g., BRA3, means sample of BRA cultivar with 3 days of PPD). Statistical analyses were made separately for each cultivar and each compound found.

Figure 1


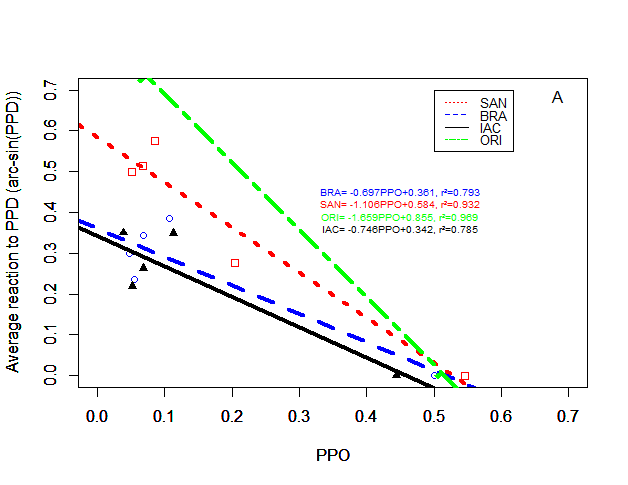


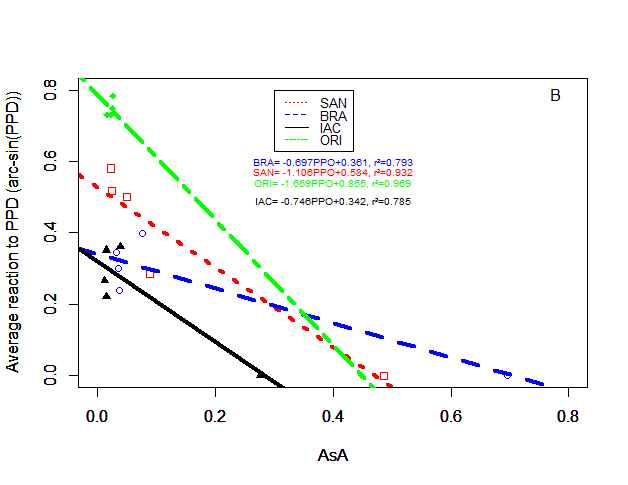


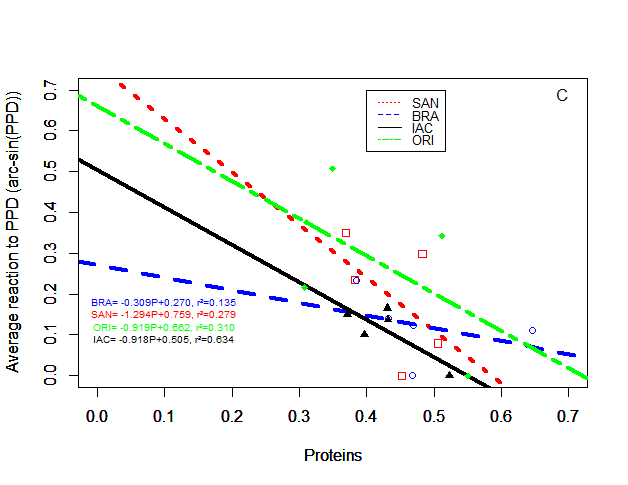


Figure 2. Changes in Scopoletin (BRA cultivar)

Scopoletin

Figure 3


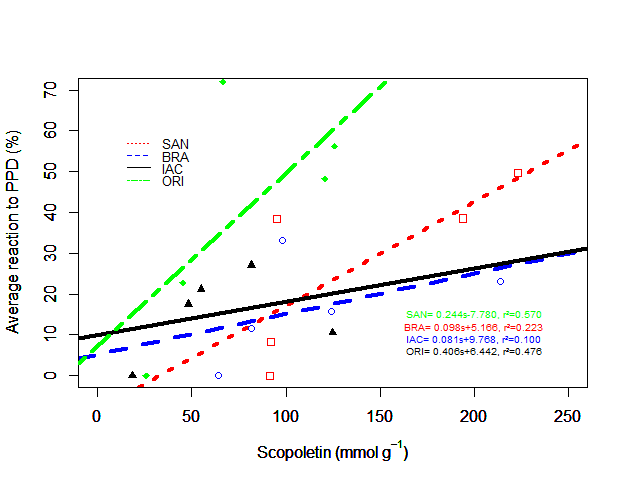


4. Changes in Soluble Sugars (BRA cultivar)

Fructose

Glucose

Saccharose

Raffinose

Figure 5: Changes in Organic acids (BRA cultivar)

D-Malic

Fumaric

Succinic

Phytic acid

Table 1

| Cultivar | Days after harvest | ^*^Mean PPD scores | Standard deviation |
| --- | --- | --- | --- |
| SAN | 0 | 0,00 | 0,0 |
| SAN | 3 | 1,57 | 0,8 |
| SAN | 5 | 7,07 | 1,4 |
| SAN | 8 | 8,93 | 1,6 |
| SAN | 11 | 10,00 | 1,6 |
| BRA | 0 | 0,00 | 0,0 |
| BRA | 3 | 3,49 | 1,4 |
| BRA | 5 | 4,64 | 2,0 |
| BRA | 8 | 6,86 | 1,8 |
| BRA | 11 | 8,15 | 1,3 |
| ORI | 0 | 0,00 | 0,0 |
| ORI | 3 | 5,99 | 1,6 |
| ORI | 5 | 8,71 | 1,8 |
| ORI | 8 | 10,00 | 1,3 |
| ORI | 11 | 10,00 | 1,0 |
| IAC | 0 | 0,00 | 0,0 |
| IAC | 3 | 3,15 | 1,0 |
| IAC | 5 | 4,93 | 1,6 |
| IAC | 8 | 6,72 | 1,6 |
| IAC | 11 | 9,10 | 1,3 |

^*^Mean of PPD scoring of five independent evaluations (harvests) and standard deviations. From each evaluation, 3 sliced cassava roots from each cultivar were sampled and evaluated to their degree of deterioration

Table 2

| **Sample^*^** | **Raffinose** | **Sucrose** | **Glucose** | **Fructose** | **Total** |
| --- | --- | --- | --- | --- | --- |
| **BRA** | **4.79b** | **112.10a** | **55.91d** | **54.19c** | **226.99e** |
| **BRA3** | **5.47a** | **91.48b** | **87.30c** | **81.25b** | **265.50b** |
| **BRA5** | **3.04c** | **25.52d** | **117.85a** | **125.74a** | **272.16a** |
| **BRA8** | **2.61cd** | **26.20d** | **102.35b** | **117.27a** | **248.43c** |
| **BRA11** | **2.54d** | **28.20cd** | **88.16c** | **111.09a** | **229.99d** |
| **ORI** | **2.69a** | **48.51b** | **15.48c** | **13.69c** | **80.37b** |
| **ORI3** | **2.86a** | **58.02a** | **30.23a** | **26.07b** | **117.18a** |
| **ORI5** | **1.70b** | **10.40c** | **25.22b** | **31.62a** | **68.94c** |
| **ORI8** | **0.00c** | **4.63d** | **17.09c** | **23.28b** | **45.01d** |
| **ORI11** | **0.00c** | **3.23d** | **9.40d** | **13.41c** | **26.05e** |
| **SAN** | **3.27a** | **48.85a** | **35.94c** | **26.06b** | **114.12c** |
| **SAN3** | **2.86a** | **49.20a** | **29.86c** | **26.78b** | **108.69c** |
| **SAN5** | **2.25b** | **13.48b** | **67.11a** | **67.94a** | **150.79a** |
| **SAN8** | **0.00c** | **5.24c** | **55.60b** | **62.41a** | **123.24b** |
| **SAN11** | **0.00c** | **4.08c** | **18.14d** | **18.40c** | **40.62d** |
| **IAC** | **3.96a** | **92.27a** | **82.26b** | **69.87c** | **248.37a** |
| **IAC3** | **3.47b** | **62.05b** | **45.74c** | **42.72d** | **153.98d** |
| **IAC5** | **3.05c** | **36.77c** | **82.08b** | **84.25b** | **206.15c** |
| **IAC8** | **2.76c** | **19.49d** | **91.77a** | **99.80a** | **213.81b** |
| **IAC11** | **0.00d** | **5.71e** | **42.23c** | **44.65d** | **92.59e** |

^*^HPLC sugar content (mg g^-1^) in cassava roots during PPD. Numbers after cultivar name means days of PPD (e.g., BRA3 means sample of BRA cultivar with 3 days of PPD). Statistical analyses were made separately for each cultivar and each compound found. Different letters between column for each cultivar means significant differences in sugar content during PPD (n=3) for the referred compound (TukeyHSD test, p<0.05).

Table 3

| **Sample^*^** | **D-Malic acid (mg g^-1^)** | **Succinic acid (mg g^-1^)** | **Fumaric acid (mg g^-1^)** |
| --- | --- | --- | --- |
| **BRA** | **0.50c** | **4.04b** | **0.86a** |
| **BRA3** | **1.41a** | **2.93c** | **0.62b** |
| **BRA5** | **0.71b** | **3.94b** | **0.04c** |
| **BRA8** | **0.44c** | **4.77a** | **0.05c** |
| **BRA11** | **0.09d** | **3.92b** | **0.04c** |
| **ORI** | **0.06b** | **2.26b** | **2.90a** |
| **ORI3** | **0.03b** | **4.38a** | **2.92a** |
| **ORI5** | **0.03b** | **2.70b** | **1.90b** |
| **ORI8** | **0.03b** | **2.25b** | **1.27c** |
| **ORI11** | **0.29a** | **2.03b** | **0.77c** |
| **SAN** | **0.29a** | **3.14c** | **1.72a** |
| **SAN3** | **0.19b** | **4.12b** | **1.36b** |
| **SAN5** | **0.13b** | **4.71b** | **0.96c** |
| **SAN8** | **0.08c** | **5.52a** | **0.83c** |
| **SAN11** | **0.17b** | **2.93c** | **0.31d** |
|  |  |  |  |
| **IAC** | **0.14a** | **4.41bc** | **0.82c** |
| **IAC3** | **0.10a** | **5.43a** | **1.29b** |
| **IAC5** | **0.08b** | **4.58b** | **0.99b** |
| **IAC8** | **0.03c** | **4.86b** | **0.82c** |
| **IAC11** | **0.08b** | **3.92c** | **6.80a** |

^*^HPLC organic acid content (mg g^-1^) in cassava roots during PPD. Numbers after cultivar name means days of PPD (e.g., BRA3 means sample of BRA cultivar with 3 days of PPD). Statistical analyses were made separately for each cultivar and each compound found. Different letters between column for each cultivar means significant differences in sugar content during PPD (n=3) for the referred compound (TukeyHSD test, p<0.05).
